# High level of telomerase RNA gene expression is associated with chromatin modification, the ALT phenotype and poor prognosis in liposarcoma

**DOI:** 10.1038/sj.bjc.6604328

**Published:** 2008-04-15

**Authors:** C J Cairney, S F Hoare, M-G Daidone, N Zaffaroni, W N Keith

**Affiliations:** 1 Centre for Oncology and Applied Pharmacology, University of Glasgow, Cancer Research UK Beatson Laboratories, Garscube Estate, Switchback Road, Bearsden, Glasgow G61 1BD, UK; 2 Unit 10, Department of Experimental Oncology and Laboratories, Fondazione IRCCS Istituto Nazionale dei Tumori, Via Venezian, 1, Milan 20133, Italy

**Keywords:** chromatin, telomerase, hTR, hTERT, ALT, liposarcoma

## Abstract

Telomere length is maintained by two known mechanisms, activation of telomerase or alternative lengthening of telomeres (ALT). The ALT pathway is more commonly activated in tumours of mesenchymal origin, although the mechanisms involved in the decision of a cell to activate either telomerase or ALT are unknown at present and no molecular markers exist to define the ALT phenotype. We have previously shown an association between chromatin remodelling, telomerase gene expression and ALT in cell line models. Here, we evaluate these findings and investigate their prognostic significance in a panel of liposarcoma tissue samples to understand the biology underlying the ALT phenotype. Liposarcoma samples were split into three groups: telomerase positive (Tel+); ALT positive; ALT−/Tel−. Differences in telomerase gene expression were evident between the groups with increased expression of hTR in ALT and Tel+ compared to ALT−/Tel− samples and increased hTERT in Tel+ samples only. Investigation of a small panel of chromatin modifications revealed significantly increased binding of acetyl H3 in association with hTR expression. We confirm that the presence of the ALT phenotype is associated with poor prognosis and in addition, for the first time, we show a direct association between hTR expression and poor prognosis in liposarcoma patients.

The activity of the ribonucleoprotein complex telomerase is reliant on the expression of both hTR and hTERT genes, the regulation of which is tightly coordinated on multiple levels by transcriptional, post-transcriptional (Zhao *et al*, 2000, 2003, 2005; Cong *et al*, 2002; Anderson *et al*, 2006; Bilsland *et al*, 2006) and epigenetic mechanisms (Atkinson *et al*, 2005; Serakinci *et al*, 2006). In most normal somatic cells, telomerase is inactivated following development so that telomeres shorten after each round of replication until they reach a critical length, signalling senescence or cell death. The majority of human tumours reactivate telomerase to maintain telomere length allowing them to bypass senescence and continue to proliferate. However, telomere length maintenance can also be achieved in some tumour types in the absence of telomerase activity through an alternative recombination-based mechanism termed ALT (alternative lengthening of telomeres) (Bryan *et al*, 1995, 1997). Alternative lengthening of telomeres is characterised phenotypically by heterogeneous telomeres, ranging from less than 3 kb to more than 50 kb in length (Henson *et al*, 2002), and the presence of ALT-associated promyelocytic leukaemia (PML) bodies (APBs), which contain telomeric DNA and the telomere binding proteins, TRF1 and TRF2, in addition to several other proteins associated with DNA recombination and repair, including RAD51 and RAD52 (Yeager *et al*, 1999; Henson *et al*, 2002).

The ALT pathway is detected in some tumours of epithelial origin, including carcinomas of the breast, lung and kidney; however, it is found at higher frequency in tumours of mesenchymal origin, such as liposarcomas, osteosarcomas and glioblastomas (Henson *et al*, 2002). This is thought to be due to tighter repression of telomerase expression in mesenchymal tissues, which have a slower cell turnover and less telomere shortening than many epithelial tissues (Henson *et al*, 2002). Several studies have highlighted an association of the ALT phenotype with sarcomas that have complex karyotypes with many chromosomal losses and gains and evidence of anaphase bridge formation, more consistent with chromosomal instability, while those that have generally more simple karyotypes, including fusion genes created from chromosomal translocations, are associated with telomerase activity (Montgomery *et al*, 2004; Ulaner *et al*, 2004).

Liposarcomas are among the most common soft tissue sarcomas forming around 20% of all mesenchymal tumours (Sandberg, 2004). They are a diverse group of mesenchymal malignancies that show multiple telomere maintenance mechanisms (TMMs) with telomerase activity and the ALT phenotype detected in a similar number of cases (Johnson *et al*, 2005; Costa *et al*, 2006). Alternative lengthening of telomeres is more frequent in well-differentiated and dedifferentiated forms (Costa *et al*, 2006), which show complex karyotypic rearrangements including supernumerary ring, giant chromosome formation and amplification (Sandberg, 2004), while telomerase activity is more frequent in myxoid forms (Costa *et al*, 2006) characterised by the t(12;16)(q13;p11) translocation (Sandberg, 2004). In addition, a significant proportion appears to show no evidence of any known TMM (Johnson *et al*, 2005; Costa *et al*, 2006). This complexity makes liposarcoma a good model system to investigate the regulation of TMMs.

The presence of any TMM, in comparison to tumours that express neither telomerase nor ALT, is associated with poor prognosis in a number of tumours of mesenchymal origin (Sanders *et al*, 2004; Costa *et al*, 2006). In osteosarcoma, telomerase expression is associated with shorter progression-free survival (Sanders *et al*, 2004), while in liposarcoma the ALT phenotype is associated with a lower survival rate (Costa *et al*, 2006). This appears to be tissue specific, as the presence of the ALT phenotype in patients with glioblastoma multiforme was associated with a longer survival rate than those with telomerase activity or no TMM (Hakin-Smith *et al*, 2003).

The molecular mechanisms involved in the decision to activate either telomerase or the ALT pathway within a particular cell type are so far unknown. As mentioned above, the presence of APBs is a defining characteristic of the ALT phenotype. A recent study showed the requirement of PML, TRF1, TRF2, TIN2, RAP1 and the MRN complex proteins MRE11, RAD50 and NBS1 for APB formation in ALT cells. The close association of APB formation with ALT highlights these genes as candidate markers or regulators of the ALT pathway (Jiang *et al*, 2007); however, the mechanisms of regulation remain to be fully determined. We have recently shown that chromatin remodelling at the telomerase gene promoters is associated with a lack of expression of hTR and hTERT in some ALT cell lines, highlighting one potential mechanism for regulating the activation of ALT or telomerase (Atkinson *et al*, 2005). Here, we analyse telomerase gene expression and the association of a small panel of chromatin modifications, with expression of these genes in liposarcoma samples with relation to TMM and patient prognosis, to investigate the molecular regulation of the ALT phenotype in tumour tissue. The identification of molecular regulators or markers of the ALT pathway will be useful not only as prognostic indicators of patient survival but may also highlight areas for therapeutic intervention to treat these tumours more effectively.

## MATERIALS AND METHODS

### Study population

Samples from 27 liposarcomas, all from adult patients treated with curative intent at the Istituto Nazionale Tumori of Milan from December 1986 to November 2003 were available for TMM, telomerase gene expression and chromatin modification analyses. These specimens represent a subset randomly selected from a larger case series already characterised for TMM (Costa *et al*, 2006). There were 13 women and 14 men, with a median age of 58 years (range, 38–91). Four patients presented at Istituto Nazionale Tumori of Milan with primary disease and 23 with recurrent tumours (22 recurrences and 1 metastasis), and they underwent different surgical procedures according to disease presentation. Tumour characteristics in terms of histology and grade are reported in Table 1. Postoperative treatment was given when there were clinical grounds for concluding that a high risk of recurrence existed: eight patients were submitted to radiotherapy, two to chemotherapy, and one to radiochemotherapy according to the treatment protocols of the multidisciplinary Istituto Nazionale Tumori of Milan Soft Tissue Sarcoma Group. The median follow-up for the entire group as of December 2006 was 108 months. During the follow-up period, 12 patients died of liposarcoma-related causes, 5 were alive with disease and 7 were disease free. Three patients were lost to follow-up.

All patients provided written informed consent to donate the tissue that was left over after diagnostic procedures were completed to the Istituto Nazionale Tumori of Milan.

### Detection of APBs and telomerase activity

Tumour tissue was sampled by a pathologist at the time of surgery and fresh-frozen. Detection of APBs by combined PML immunofluorescence and telomere fluorescence *in situ* hybridisation (FISH) and telomerase activity by the telomeric-repeat amplification protocol (Kim *et al*, 1994) with the TRAPeze kit (Intergen Company, Oxford, UK) were carried out as outlined in Costa *et al* (2006).

### Sample preparation for RNA extraction and ChIP assay

Frozen tissue samples varying in size from 50 to 120 mg were disrupted into small pieces with liquid nitrogen and a mortar and pestle and then split in half for use in chromatin immunoprecipitation (ChIP) assay and RNA extraction for telomerase gene expression analysis.

### Expression analysis

Further disruption of the tissue was carried out in RA1 buffer plus *β*-mercaptoethanol (Nucleospin II RNA extraction kit, Macherey-Nagel, Düren, Germany) in a Ribolyser (Hybaid, Waltham, MA, USA) at a setting of 5.5 for 5 × 10 s pulses with 30 s pauses between. Samples were then passed through an 18-gauge needle to shear genomic DNA. RNA was extracted using the Nucleospin II RNA extraction kit (Macherey-Nagel) following the manufacturer's instructions. hTR expression was analysed by quantitative PCR (Q-PCR) as described previously (Atkinson *et al*, 2005) and taken as a percentage of the riboprotein S15 (primers (RETROscript kit, Ambion) S15 Forward TTCCGCAAGTTCACCTACC, S15 Reverse CGGGCCGGCCATGCTTTACG). hTERT expression was analysed by semiQ-PCR using primers designed to detect the four main hTERT splice variants (Atkinson *et al*, 2005). Samples were adjusted for S15 expression prior to splice variant PCR, and total hTERT levels were taken as the sum of all splice variant products quantified using the Agilent Bioanalyser as described previously (Keith and Hoare, 2004).

### Chromatin immunoprecipitation

Chromatin immunoprecipitation assays were carried out using the Upstate Biotechnologies protocol as described previously (Atkinson *et al*, 2005), with some modifications for tissue handling adapted from Farnham's ChIP protocol for tissues (http://genomics.ucdavis. edu/farnham/). Half of the disrupted tumour sample was fixed in PBS containing 1% formaldehyde. After 10 min, 0.125 M glycine was added to quench the formaldehyde and stop the cross-linking. Tissue was rinsed in cold PBS and further disrupted in a dounce homogeniser. Cells were lysed in Farnham's cell lysis buffer (5 mM PIPES, pH 8.0; 85 mM KCl; 0.5% NP40; 1 mM PMSF; 2 *μ*g ml^−1^ aprotonin; 1 *μ*g ml^−1^ pepstatin) using 500 *μ*l per 50 mg tissue, then passed through a series of needles (18–25 gauge) to release the nuclei. Samples were resuspended in SDS lysis buffer (Upstate Biotechnologies, Watford, UK) with protease inhibitors (400 *μ*l buffer per 50 mg tissue) and incubated on ice for 90 min. The remainder of the protocol was carried out as described (Atkinson *et al*, 2005); however, no DNA purification was required.

### Quantitative PCR

Products from ChIP assays were analysed by Q-PCR as described previously (Atkinson *et al*, 2005). Data are presented as a percentage of input following subtraction of the background pulled down with the no antibody control.

### Antibodies

Antibodies used were AcH3, AcH4 and TriMeK20 (all Upstate Biotechnologies) and TriMeK9 (Abcam, Cambridge, UK). Tissue (13–25 mg) was required per antibody.

### Data analysis

Criteria used to define the presence of telomerase and ALT phenoypes were previously detailed (Costa *et al*, 2006). The cutoff to discriminate between low and high hTR expression was defined as 97% of S15 expression (mean of all samples minus two standard errors). hTERT-positive cases were defined as those expressing the wild-type variant. *In vitro* measurements were performed by personnel blinded to patient data and clinical outcome, whereas clinical data were collected by personnel blinded to *in vitro* data. Kruskal–Wallis, Mann–Whitney and *χ*^2^ tests were used to assess differences in the distribution of data categorised according to the investigated features. The clinical end point of this study was cause-specific mortality, and the time of its occurrence was computed from the date of first diagnosis to the time of death or censored at the date of the last recorded follow-up for living patients or for those who died from liposarcoma-unrelated conditions. Survival analysis was carried out with Cox regression models after checking for the proportional hazard assumption. SAS software (SAS Institutes Inc., Cary, NC, USA) was used for statistical calculations. All *P*-values were two-sided; *P*<0.05 was considered to be statistically significant.

## RESULTS

### How does telomerase gene expression relate to the ALT phenotype?

A total of 27 liposarcoma tissue samples were collected from 27 different patients and evaluated for telomerase activity using the TRAP assay and for the ALT phenotype using immunofluorescence/FISH to detect ALT-associated PML bodies. The samples were then split into three groups depending on TMM status: telomerase positive (Tel+); ALT positive; both Tel−/ALT−. To explore the molecular mechanisms underlying the morphological determination of the ALT phenotype, we analysed expression of hTR and hTERT at the RNA level.

**Figure 1:**
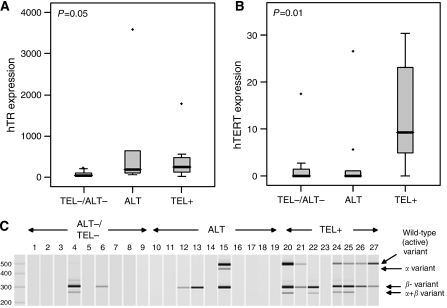
Telomerase gene expression in liposarcoma samples. (**A**) hTR expression taken as a percentage of the riboprotein S15; (**B**) total hTERT expression in ng per *μ*l. The grey box defines 25 and 75% quartiles, while error bars represent the first and 99th percentiles of the distribution. Dots represent outliers and the black line defines the median of the distribution. *P*-values were calculated using the Kruskal–Wallis test, which tests the likelihood of all medians being the same. (**C**) Four major hTERT splice variant PCR products were quantified using the Agilent Bioanalyser.

hTR expression, normalised to expression of the riboprotein S15, was determined by Q-PCR and expressed as a percentage of S15 (Figure 1A). All of the liposarcoma samples evaluated in this study expressed hTR to varying degrees; however, significant differences in expression were found within the three groups (*P*=0.05). Samples that were negative for both telomerase and ALT had mostly low-level expression with a median level of 48% of S15 expression (range 20–333%), while those that were Tel+ had an even distribution of expression with a median level of 242% (range 31–562% with one outlier at 1783%). Those samples expressing the ALT phenotype, on the other hand, appeared to have a higher distribution of expression, although the median value of 199% did not reflect this (range 71–653% with one outlier at 3575%).

hTERT expression evaluated by semi-Q-PCR is shown in Figure 1B. The majority of liposarcoma samples, which were negative for both telomerase and ALT or expressed the ALT phenotype, had little or no total hTERT expression. The median level for both of these groups was 0 ng *μ*l^−1^. Conversely, the majority of samples that were Tel+ expressed significantly higher levels of hTERT in comparison to the other groups, with a median level of 9.4 ng *μ*l^−1^. TERT expression is known to be regulated by differential splicing; therefore, we investigated the expression pattern of the four main splice variants of hTERT (Figure 1C). Little expression of any of the splice variants was detected in the samples, which were negative for both telomerase and ALT. The inactive beta variant was detected in two of these samples and the active wild-type variant was not detected at all. Likewise, for samples expressing the ALT phenotype, three samples expressed the inactive beta variant and only one sample expressed the active wild-type variant. It is of interest to note that this sample is the only one in which the dominant-negative alpha variant is detected and it is one of only two ALT samples expressing no hTR. In contrast, the active wild-type variant could be detected in the majority of the Tel+ samples with the exception of samples 22 and 23. Lack of the wild-type variant in these samples may reflect differences in sensitivity between the TRAP assay and the splice variant PCR.

Table 2 provides a summary of the expression data generated for the liposarcoma samples separated by phenotype and expression, where hTR+ is taken as over 97% of S15 expression (mean of all samples minus two standard errors) and hTERT+ is taken as expressing the wild-type splice variant. A statistically significant (*P*=0.004) association was observed between the absence or presence of TMMs and telomerase gene expression. Telomerase enzymatic activity is dependent on expression of both hTR and hTERT; therefore, it is interesting to note that the only samples positive for both hTR and hTERT expression lie within the Tel+ group. The fact that two other samples lie within the Tel+ group, although they appear to be negative for hTERT or both hTERT and hTR, could reflect differences in assay sensitivity rather than phenotype, as mentioned previously. The majority of samples expressing the ALT phenotype, on the other hand, express only hTR, with the exception of one sample that expresses hTERT alone and two more that have neither hTERT nor hTR expression. A high level of hTR and little or no hTERT expression in association with the ALT phenotype has been shown previously in cell line models (Hoare *et al*, 2001; Atkinson *et al*, 2005). Finally, samples lacking both telomerase expression and ALT are negative for hTR and hTERT or express only hTR.

### How does the chromatin landscape relate to telomerase gene expression in liposarcoma?

**Figure 2:**
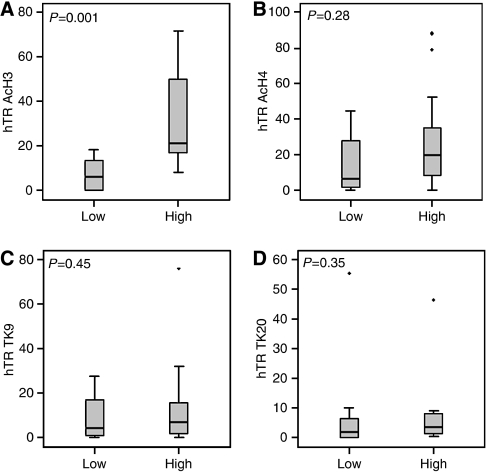
Chromatin modifications associated with low and high hTR expressing liposarcoma samples represented as a percentage of input chromatin. (**A**) Association of acetyl histone H3; (**B**) association of acetyl histone H4; (**C**) association of tri-methyl K9 of H3; (**D**) association of tri-methyl K20 of H4. The grey box defines 25 and 75% quartiles, while error bars represent the first and 99th percentiles of the distribution. Dots represent outliers and the black line defines the median of the distribution. *P*-values were calculated using the Mann–Whitney test, which tests the likelihood of all medians being the same.

**Figure 3:**
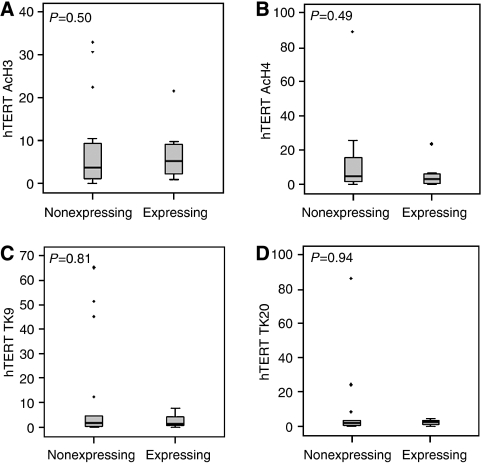
Chromatin modifications associated with liposarcoma samples expressing wild-type hTERT or not represented as a percentage of input chromatin. (**A**) Association of acetyl histone H3; (**B**) association of acetyl histone H4; (**C**) association of tri-methyl K9 of H3; (**D**) association of tri-methyl K20 of H4. The grey box defines 25 and 75% quartiles, while error bars represent the first and 99th percentiles of the distribution. Dots represent outliers and the black line defines the median of the distribution. *P*-values were calculated using the Mann–Whitney test, which tests the likelihood of all medians being the same.

It is well known that gene expression can be controlled at the level of chromatin and we have previously shown that telomerase gene expression is subject to epigenetic regulation by chromatin remodelling in cell line models. To determine whether modification of the chromatin landscape bears any relation to telomerase gene expression in liposarcoma, we used chromatin immunoprecipitation to study the association of a small panel of histone modifications with the telomerase gene promoters. Samples were grouped according to levels of hTR expression or by the presence or absence of the hTERT wild-type splice variant. The association of two modifications related to active gene expression, acetyl histone H3 and acetyl histone H4, and two modifications related to gene repression and the heterochromatic state, tri-methyl lysine 9 of histone H3 and tri-methyl lysine 20 of histone H4, with the hTR and hTERT promoters were investigated within these expression groups (Figures 2 and 3, respectively).

The data in Figure 2 show a relationship between hTR expression and acetylation of histones H3 and H4. Association of acetyl H3 (Figure 2A) with the hTR promoter is significantly increased as a function of hTR expression (median levels of 5.5 and 20.8% of input for low and high expressors, respectively; *P*=0.001), while the increase in association of acetyl H4 with the hTR promoter in high expressors compared to low expressors (median levels of 19.4 and 6.3% of input, respectively) did not reach significance (*P*=0.28). In the case of the negative modifications, no significant differences in association were seen between high and low expressing samples (Figure 2C and D). These results suggest that hTR expression in these liposarcoma samples is associated with positively acting histone modifications and that the negative modifications we have chosen for this study bear no relation to hTR gene expression.

The relationship between binding of these chromatin modifications and hTERT expression is less obvious, as there are no significant differences between those samples expressing the wild-type variant and those that do not (Figure 3). Although the small differences that we do see in association of these modifications appear uninformative and are not statistically significant, the actual levels of hTERT expression are very low with normal levels of 1–30 copies of the gene per cell (Yi *et al*, 2001); therefore, these slight differences in chromatin occupancy could be biologically significant. It could be that for hTERT the lack of negatively acting chromatin modifications is more important than the presence of positive modifications, at least of those chosen for this study.

### Does chromatin modification at the telomerase gene promoters distinguish between the differential expression of the three phenotypes?

The results of the previous section suggest that while acetylation of histones at the hTR promoter was related to hTR expression levels, no clear pattern of regulation could be seen for hTERT. To further study the mechanism of regulation of the TMM phenotype, we next looked at the potential of these chromatin modifications as molecular markers to distinguish among the three phenotype groups within our liposarcoma samples. Samples were grouped according to telomerase status or ALT phenotype following chromatin immunoprecipitation as previously.

**Figure 4:**
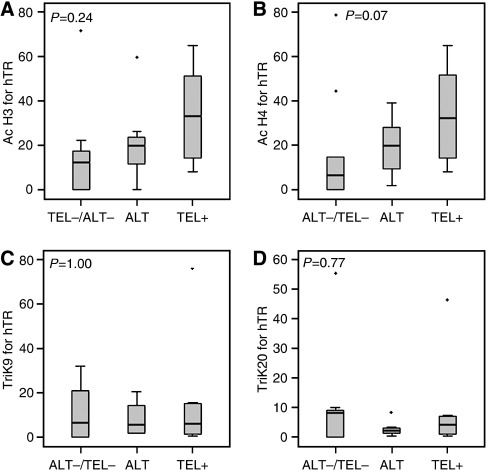
Chromatin modifications at the hTR promoter in samples grouped by phenotype represented as a percentage of input chromatin. (**A**) Association of acetyl histone H3; (**B**) association of acetyl histone H4; (**C**) association of tri-methyl K9 of H3; (**D**) association of tri-methyl K20 of H4. The grey box defines 25 and 75% quartiles, while error bars represent the first and 99th percentiles of the distribution. Dots represent outliers and the black line defines the median of the distribution. *P*-values were calculated using the Kruskal–Wallis test, which tests the likelihood of all medians being the same.

Figure 4 shows association of chromatin modifications at the hTR promoter within the three phenotype groups. Although none of the differences in median levels reached statistical significance, clear trends were seen in the association of positively and negatively acting chromatin modifications across the three phenotype groups. Consistent with expected results, the highest levels of the positively acting marks, acetylation of H3 (Figure 4A) and H4 (Figure 4B), were found in Tel+ samples (median levels of 32.3 and 23.7% of input, respectively), which also have the highest median levels of hTR expression. No clear association was observed between the levels of the negatively acting marks, tri-methyl K9 (Figure 4C) and tri-methyl K20 (Figure 4D) and the distinct TMM subsets. As was the case with expression levels for hTR, levels of acetylation in those samples expressing the ALT phenotype lie somewhere between those those were either positive for telomerase or negative for both telomerase and ALT (Figure 4A and B).

In agreement with hTERT expression, the results for association with the hTERT promoter are not so clear and no obvious relationships between binding of the chosen chromatin modifications and telomerase status or ALT phenotype were found (data not shown).

### How do telomerase gene expression and the ALT phenotype relate to clinical outcome in patients with liposarcoma?

**Figure 5:**
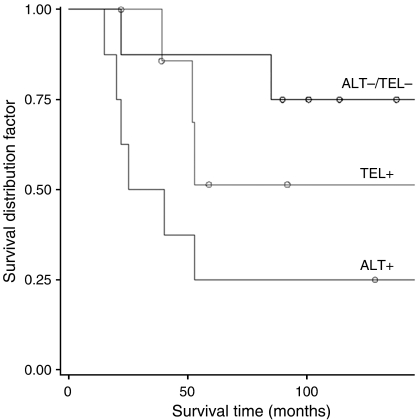
Overall survival as a function of TMM. Comparison between patients with ALT−/Tel− tumours and those with telomerase-positive or ALT tumours.

To determine whether telomerase gene expression and the ALT phenotype would bear any relationship to patient survival, the clinical outcome was evaluated at the median time of follow-up, which in the present series was 9 years. The presence or absence of TMMs segregated liposarcoma patients into distinct prognostic subsets (Figure 5): in fact, 9-year probability of survival was 75% for the 9 patients with ALT−/Tel− tumours, 51% for the 8 patients with telomerase-positive tumours and only 25% for the 10 patients with ALT-positive tumours. Specifically, compared to patients with ALT-positive tumours, those with ALT−/Tel− or with telomerase-positive tumours had a 0.19-fold (95% confidence limit (CL), 0.04–0.94, *P*=0.042) and a 0.38-fold (95% CL, 0.10–1.53, *P*=0.17) hazard of death. However, when adjusted for tumour grade, the lower hazard of death for patients with ALT−/Tel− tumours *vs* those with ALT-positive tumours became only suggestive of statistical significance (hazard ratio (HR), 0.15; 95% CL, 0.016–1.48, *P*=0.09).

Tumour grade and the histological subtype of the liposarcoma samples are important considerations that should be taken into account when looking at the relationship between telomerase gene expression, TMM and clinical outcome. Consistent with previous results (Costa *et al*, 2006), clinical outcome and TMM correlate with histological subtype. Those in ALT or Tel-positive groups, which have a poorer prognosis, have an increased incidence of the more aggressive liposarcoma subtypes, dedifferentiated or usual myxoid and round-cell myxoid subtypes, respectively, while the well-differentiated tumours were the most abundant subtype within the ALT−/Tel− group, which has the best prognosis (summarised in Table 3). Similarly, taking into account tumour grade, a statistically significant association (*P*=0.016; *χ*^2^ test) was observed between TMM and grade. Grade 1 tumours were mainly represented by ALT−/Tel− cases (7/12), grade 2 by Tel-positive (5/9) and grade 3 by ALT-positive (5/6) (reported in Table 1).

**Figure 6:**
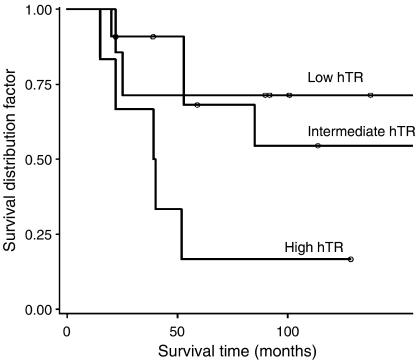
Overall survival as a function hTR expression. Comparison among patients with tumours expressing low, intermediate and high hTR levels. High level of telomerase RNA classes were defined on the basis of its frequency distribution, using the first and the third quartiles as cutoff values.

The results of this study suggest that levels of telomerase gene expression can distinguish among the three TMM phenotype groups; therefore, the relationship of hTR and hTERT expression to prognosis was considered in two ways, by taking the liposarcoma samples as one whole group or by subdividing this large group by the presence or absence of the ALT phenotype. Firstly, we looked at the prognostic significance of hTR and hTERT in all liposarcoma samples. When considered as dichotomous variables, using the mean of all samples minus two standard errors (97% of S15 expression), as a cutoff to define low or high hTR expression, or any detectable level of wild-type variant for hTERT, no association was observed between these two variables and survival (HR and 95% CLs for hTR, 1.65 and 0.44–6.24, *P*=0.46; HR and 95% CL for hTERT, 1.57 and 0.45–5.47, *P*=0.48). Conversely, when hTR abundance was considered as a continuous variable, a trend in favour of a worse prognosis for patients whose tumours expressed the higher levels was observed (*P*=0.067, Figure 6), which reached statistical significance after logarithmic transformation (HR and 95% CL, 1.65 and 1.03–2.65, *P*=0.036). In contrast, hTERT expression failed to provide prognostic information, even when considered as a continuous variable.

We next looked at the prognostic significance of hTR and hTERT adjusted for the most important variable in this series, the presence or absence of ALT. While hTERT expression gave no prognostic information, hTR expression had some prognostic significance within ALT− (HR and 95% CL, 2.15 and 0.97–6.52, *P*=0.058), but not within ALT+ subsets.

The association between high hTR expression and poor prognosis led us to investigate any relationship between hTR expression and histological subtype or tumour grade. When considered as a dichotomous variable, using the mean of all samples minus two standard errors as the cutoff, no significant association was shown between hTR expression and histological subtype or tumour grade of the liposarcoma samples. However, when the median expression levels were considered, we found a trend in association, with the lowest hTR expression in those tumours of the well-differentiated subtype compared to the other more aggressive subtypes (summarised in Table 3). Similarly, a significantly higher hTR expression (*P*=0.0136; Kruskal–Wallis test) was observed in grade 3 tumours (median value, 601) compared to grade 2 (median value, 116) or grade 1 tumours (median value, 100).

Overall, from these data, it would appear that the presence of the ALT phenotype and high levels of hTR expression are singly associated with poor prognosis in liposarcoma patients.

## DISCUSSION

In this study, we sought to investigate the mechanisms regulating the ALT phenotype in liposarcoma tissue samples, with specific emphasis on telomerase gene regulation, to determine how the morphological definition of ALT relates to the underlying biology and clinical outcome. Significant differences in the expression of hTR were found among the three phenotype groups, with samples positive for telomerase or ALT having higher expression levels than those that were negative for both telomerase and ALT. Expression of hTERT, on the other hand, was significantly increased only in Tel+ samples.

Liposarcoma samples were initially separated according to their TMM using telomerase activity and the presence or absence of APBs. At present, we have little indication of how these TMMs are regulated at the molecular level to determine which is activated in particular tumour types. We have previously shown in cell line models that regulation of telomerase gene expression is subject to control at the epigenetic level. Methylation of CpG islands within the hTR promoter in several ALT cell lines was associated with gene repression (Hoare *et al*, 2001), while remodelling of the chromatin environment in the promoters of both hTR and hTERT was associated with differential gene expression in Tel+ or ALT positive tumour cell lines and normal cell line controls (Atkinson *et al*, 2005) and repression in human mesenchymal stem cells (Serakinci *et al*, 2006). Furthermore, treatment of Tel− cell lines with the histone deacetylase inhibitor Trichostatin A and the demethylating agent 5-aza 2′deoxycytidine induced expression of hTR and hTERT, consistent with decreased methylation and increased association of acetylated histones at these gene promoters (Atkinson *et al*, 2005; Serakinci *et al*, 2006). To evaluate these cell line data in clinical samples, we investigated chromatin remodelling in liposarcoma tissue samples using a limited panel of histone modifications associated with gene activation and repression. An increased binding of positively acting modifications, acetylation of histone H3 and histone H4, was shown in association with higher levels of hTR expression, while no significant differences were shown in association with hTERT expression. In addition, no significant alterations in the binding of the negatively acting modifications, tri-methyl lysine 9 of histone H3 and tri-methyl lysine 20 of histone H4, at the telomerase gene promoters were associated with either hTR or hTERT expression. The association between histone acetylation and hTR expression has been shown previously in cell line models; however, this is the first time that the correlation between histone modification and gene expression has been demonstrated in clinical samples.

It has been shown that APBs are not always associated with the ALT phenotype (Fasching *et al*, 2005) and although candidate genes have been suggested (Jiang *et al*, 2007), no molecular markers for the detection of ALT have yet been confirmed. Previous work in cell line models highlighted an association of tri-methyl K20 of histone H4 with both telomerase gene promoters in ALT cell lines, suggesting that chromatin modifications may have potential as novel markers of the ALT phenotype (Atkinson *et al*, 2005). We investigated the potential for our limited panel of chromatin modifications, in association with the telomerase gene promoters, to distinguish among the three phenotype groups. Although no significant differences in chromatin remodelling were found among the three groups, trends of association were shown with acetyl H3 and H4 at the hTR promoter. Perhaps by using a more extensive panel of modifications or a genome-wide screen, other candidate molecular markers for the ALT phenotype could be uncovered in future experiments.

While we found no significant differences among the three TMM phenotype groups using the limited panel of chromatin modifications studied, differences were highlighted in telomerase gene expression itself. We further investigated expression of hTR and hTERT in relation to histological subtype, tumour grade and patient survival as potential prognostic indicators in liposarcoma. While hTERT expression was devoid of prognostic significance, statistical analysis highlighted a trend in favour of worse prognosis for patients with tumours expressing high levels of hTR. No significant association was found between hTR abundance and histological subtype or tumour grade when hTR expression was considered as a dichotomous variable; however, a trend in association of high hTR with the more aggressive subtypes and significant association of high hTR with grade 3 tumours was highlighted when median expression levels of hTR were considered. To our knowledge, this is the first time that such an association between hTR expression and poor prognosis for patients with liposarcoma has been documented.

Evidence is accumulating in support of the relationship between telomerase gene expression, TMM and prognosis. It has been shown in a number of mesenchymal tumour types that the presence of any TMM is predictive of a poorer outcome in patients (Ulaner *et al*, 2003; Sanders *et al*, 2004; Costa *et al*, 2006). The statistical analysis of our patient material with relation to TMM showed that patients with tumours expressing the ALT phenotype had the lowest survival rate at follow-up. Furthermore, those patients whose tumours neither expressed telomerase nor ALT had the highest survival rate with a lower HR for death than those expressing telomerase or the ALT phenotype, although this was only suggestive of significance when adjusted for tumour grade. Consistent with previously published results (Costa *et al*, 2006), the more aggressive histological subtypes, dedifferentiated or normal-myxoid and round-cell myxoid, were found within ALT or Tel+ groups, respectively, while the least aggressive well-differentiated liposarcomas were all within the ALT−/Tel− group. Similarly, the highest proportion of grade 3 tumours were found within the ALT group and the majority of Tel+ tumours were grade 2, while those that were ALT−/Tel− were mostly of the least aggressive grade 1. The association of the ALT phenotype with clinical aggressiveness has been shown previously for liposarcoma (Costa *et al*, 2006); however, the prognostic significance of ALT appears to be tissue and tumour type-specific. In osteosarcoma, hTERT is a predictive indicator of worse prognosis, with a trend in favour of shorter progression-free survival in patients whose tumours expressed telomerase rather than ALT (Sanders *et al*, 2004), while in glioblastoma multiforme the presence of ALT is actually indicative of a better prognosis (Hakin-Smith *et al*, 2003). The correlation between TMM, telomerase gene expression and the various tissue-specific clinical outcomes may reflect the underlying complexity of telomere biology in different tumour types and certainly is of great importance to understanding telomere maintenance and telomerase regulation.

As both expression of the ALT phenotype and high levels of hTR were predictive of poor prognosis as individual factors, we also investigated the prognostic significance of hTR expression as a function of ALT presence. While hTR expression held no prognostic significance within the ALT expressing subgroup, there was a trend in favour of a worse prognosis for patients with ALT− tumours expressing high levels of hTR.

From the results of our study and consistent with previous results (Costa *et al*, 2006), the ALT phenotype is a strong predictor of poor prognosis in liposarcoma; however, we have also shown a novel direct association between high levels of telomerase RNA gene expression and poor prognosis. This is irrespective of any particular TMM but has prognostic significance when the group of liposarcoma samples is considered as a whole, with the high hTR expressing group including some ALT, Tel+ and ALT−/Tel− samples. We are now beginning to gain some insight into the mechanisms regulating the ALT phenotype at the molecular level. While the search continues for specific markers to define ALT and to predict the clinical outcome in ALT associated tumours more effectively, the data presented here provide a platform to define the relationship between clinical outcome and the underlying biology of telomere maintenance in cancer.

## Figures and Tables

**Table 1:** Main tumour characteristics

**Tumour**	**Grade**	**Histological subtype**	**TMM**	**hTR expression**	**hTERT wild-type expression**
S1	1	WD	ALT−Tel−	Low	No
S2	1	WD	ALT−Tel−	Low	No
S3	2	WD	ALT−Tel−	High	No
S4	1	WD	ALT−Tel−	High	No
S5	1	WD	ALT−Tel−	High	No
S6	2	WD	ALT−Tel−	Low	No
S7	1	Myx	ALT−Tel−	High	No
S8	1	WD	ALT−Tel−	Low	No
S9	1	WD	ALT−Tel−	Low	No
S10	1	DD	ALT+	No result	No
S11	1	DD	ALT+	High	No
S12	3	DD	ALT+	High	No
S13	3	DD	ALT+	High	No
S14	3	RC	ALT+	High	No
S15	2	DD	ALT+	Low	Yes
S16	3	DD	ALT+	High	No
S17	3	DD	ALT+	High	No
S18	2	Myx	ALT+	Low	No
S19	1	RC	ALT+	High	No
S20	2	Myx	Tel+	High	Yes
S21	3	Myx	Tel+	High	Yes
S22	2	DD	Tel+	High	No
S23	1	RC	Tel+	Low	No
S24	2	RC	Tel+	High	Yes
S25	2	DD	Tel+	High	Yes
S26	1	Myx	Tel+	High	Yes
S27	2	RC	Tel+	High	Yes

DD=dedifferentiated; Myx=usual myxoid; RC=round-cell myxoid; WD=well differentiated.

Cutoff for low or high hTR expression was taken as the mean of all samples minus two standard errors.

**Table 2:** Summary of telomerase gene expression in relation to ALT phenotype or telomerase status

	**hTR−/hTERT−**	**hTR−/hTERT+**	**hTR+/hTERT−**	**hTR+/hTERT+**
ALT−/TEL−	5	0	4	0
ALT+	2	1	7	0
TEL+	1	0	1	6

**Table 3:** Summary of histological subtype in relation to ALT phenotype or telomerase status and hTR expression

	**Well differentiated**	**Usual myxoid**	**Round-cell myxoid**	**Dedifferentiated**
ALT−/Tel−	8	1	0	0
ALT+	0	1	2	7
Tel+	0	3	3	2
hTR % S15	48 (20–232)	212 (96–562)	271 (31–653)	393 (71–3575)
